# Customization of manual wheelchair components: a state-of-the-art review

**DOI:** 10.1186/s12984-025-01547-6

**Published:** 2025-03-24

**Authors:** Sameer More, Michelle Dunn, Shannon A. Rios, Rachael McDonald

**Affiliations:** 1 Swinburne University of Technology, John Street, Hawthorn, 3122 Melbourne, Victoria Australia; 2 The University of Melbourne, 3052 Parkville, Victoria Australia

**Keywords:** Manual wheelchair, Custom-contoured cushions, Design, Manufacture, Customization, 3D scanning, Digital workflows

## Abstract

Custom wheelchairs, tailored to users’ unique needs, have gained increasing attention because of their potential to increase mobility, comfort, and overall quality of life for the people using them. Employing a state-of-the-art review methodology, this literature review systematically categorizes the existing body of research on the design and manufacture of customizable manual wheelchair components, identifies current practices, and suggests areas for further research. We found that the literature to date has focused on the design and manufacture of custom-contoured cushions, and few studies have focused on other components or wheelchair customization as a whole. Technological advances in the past decade have seen the move from manual, time-consuming customization processes to digital workflows with the support of design tools. Advanced technologies such as 3D scanning, parametric modeling, product configuration systems, and finite element analysis have emerged as tools for enhancing the performance and aesthetics of wheelchair components. While the published literature provides a valuable foundation, the field of wheelchair customization is rapidly evolving, driven by ongoing innovations from manufacturers. This review highlights the need for further research to bridge the gap between academic knowledge and real-world progress in the design and manufacturing of custom manual wheelchairs.

## Background

People with a diverse range of issues, typically mobility impairments or other disabilities that affect their ability to walk, use manual wheelchairs as their primary form of mobility. This includes those with poor balance and movement conditions, such as lower limb amputation, individuals with spinal cord injuries, osteoarthritis, and other degenerative and neurological diagnoses [1]. Regardless of the duration of use of the wheelchair, it is important that appropriate, well-fitted wheelchairs are provided to users. The wheelchair provision guidelines by the World Health Organization (WHO) define appropriateness in terms of proper fit, postural support and effective service delivery [2]. Obtaining an appropriate wheelchair is not only a matter of user comfort and efficiency, but also a means to exercise human rights [3, 4]. If a manual wheelchair is not properly fitted to the user, it can lead to a range of negative outcomes that can significantly affect their everyday life [5]. From a physical perspective, a poorly fitting wheelchair can encourage poor posture, the development of secondary deformity and/or development of pressure ulcers [6, 7]. From a functional perspective, a poorly fitting wheelchair can have implications such as reduced ability to efficiently push a chair, resulting in shoulder injury or not being able to access work or school [8]. Customizing a wheelchair to an individual’s specific needs is essential for improving the efficiency of the wheelchair, enhancing user comfort, and mitigating negative outcomes. This customization process enables the user to participate more fully in their desired life situations, promoting greater independence, social inclusion, and overall well-being [8, 9]. However, research indicates that despite the recognized importance of appropriate wheelchair provision, this remains an ongoing challenge globally [10].

The Australian Standard (AS/NZS 3695.1:2011), which is based on the ISO 7176 standard, defines custom-made wheelchairs as “wheelchairs that are made according to a request by a health professional, who specifies the design characteristics or construction of the wheelchair and are intended to be used only in relation to a particular individual” [11]. Within this paper, custom manual wheelchairs fall under the broad category of ultralightweight wheelchairs, which weigh less than 13.6 kg and are intended for use by long-term active users [12, 13]. Recent studies with users and providers of mobility aids have indicated the need for research on advanced wheelchair technologies [14, 15]. Additionally, ways to shorten the time it takes for clients to purchase their equipment [15] and the incorporation of technologies and manufacturing techniques from other fields [16] have also been emphasized. These findings align with the recommendations in the WHO’s global report on assistive technology (AT). The WHO advises exploring innovative design and manufacturing tools while considering the possibilities of customization in the design process. This approach helps ensure that products are safe, affordable, and contextually appropriate [17].

Recent advances in materials, design, and manufacturing have made wheelchair customization easier. These include improvements in computer-aided design tools such as parametric modeling and additive manufacturing [18]. These advances make it possible to create, manufacture, and modify custom designs in a short time frame [19]. As such, this review aims to synthesize current research on the customization of manual wheelchair components, specifically in the design and manufacturing context.

## Methodology

This article presents a state-of-the-art literature review based on the methodology developed by Barry et al. [20]. The purpose of such reviews is to create a summary of current viewpoints on a topic, outline its historical developments, and propose directions for further research. To achieve this, the method follows a six-step approach: (1) determine the initial research question and field of inquiry, (2) determine the timeframe for the review, (3) finalize research questions to reflect the timeframe, (4) develop a search strategy, (5) analysis, and (6) reflexivity. Each of these steps is described below.

### Research question

There has been a growing emphasis on the importance of customizing manual wheelchairs [6], which are inherently expensive because of the manufacturing costs and coupled services that ensure a good fit [21]. An initial literature search identified several studies that looked at the design and manufacturing aspects of manual wheelchair components, but a specific focus on customized components was missing. In addition, the information was scattered, and a consolidated review was missing. Hence, the initial research question guiding this literature review is “What is the current state of design and manufacturing for customized components of manual wheelchairs.”

### Determine the timeframe

The concept of manual wheelchair customization was the result of the ultralight wheelchair revolution that took place in the late 1970s. The term ultralight was used to describe wheelchairs adapted for daily use and was originally developed for wheelchair sports [22]. This revolution culminated in great advances after the International Year of the Disabled in 1980 [23]. This can be considered the beginning of state-of-the-art thinking in wheelchair customization [22]. As a result, the timeframe for this review is from 1980 to 2024.

### Finalize the research questions to reflect the timeframe

The original research question was revised to better align with the objective of studying custom manual wheelchairs for daily use. This also led to the formulation of supplementary questions to guide the analysis process.

#### Finalized research question

What is the current state of research on the design and manufacturing of customizable components of manual wheelchairs for daily use?

#### Supplementary questions

1. What is the general trend in publications?
2. What are the main research areas?
3. What are the latest advancements in the design and manufacturing techniques used for customized wheelchair components?
4. How do digital technologies such as computer-aided design (CAD) contribute to the customization of manual wheelchairs?
5. How is the effectiveness of custom components evaluated?

### Development of a search strategy

In April 2023, an electronic search of databases, including the Scopus, Web of Science, PubMed, and IEEE databases, as well as manufacturer websites, was conducted. The search strategy is outlined below in Table 1.

The inclusion criteria for topics of interest in peer-reviewed articles were the history, necessity and benefits of wheelchair customization; and the design, manufacturing, or evaluation of custom wheelchair components. Eligible sources were journal articles, conference proceedings, books, and standards. Website browsing of some manufacturers was done to capture innovations from manufacturers. These specific manufacturers were chosen as they had information related to the design or manufacture of customizable wheelchair components. Specific sections of the website such as made to measure, custom seating, shape capture, and configurator or visualizer were browsed. The publication window considered spanned from 1980 to 2024, with the requirement that articles and websites be published in English. Additionally, citation searching within included articles was used to identify relevant articles using the same inclusion criteria as discussed above.

**Table 1:** Search strategy

Database	Search term	Search filter
PubMed	‘custom wheelchair’	All fields
IEEE	wheelchair AND (“custom” OR “bespoke” OR “made to measure”)	All fields
Web of Science, Scopus	wheelchair AND (“custom” OR “bespoke” OR “made to measure”)	Topic search
**Manufacturer websites**		
Website browsing of specific manufacturers such as TiLite wheelchairs (Permobil), RGK wheelchairs (Sunrise Medical), Quickie wheelchairs (Sunrise Medical), Kuschall wheelchairs, Progeo wheelchairs (Permobil), SHAPE Custom Contoured Seating (Ottobock), PINDOT custom seating (Invacare Corporation), PRM Rehab		

### Analyses

The Preferred Reporting Items for Systematic Reviews and Meta-Analyses (PRISMA) flowchart was used to report the screening process [24]. The web version of the Rayyan app [25] was used to assist in the screening process. An Excel spreadsheet was used for data extraction. The extracted data included (1) keywords, (2) authors, (3) research design, (4) target population, (5) country, (6) sample size, (7) data collection instruments, and (8) study findings and suggestions for future work.

### Reflexivity

The state-of-the-art review methodology is based on the relativist ontology and a subjectivist epistemology, so the point in time at which the review is conducted, as well as the experiences and expertise of the review team shape the analysis process and the conclusions drawn from the review. Therefore, we acknowledge that teams with different levels of expertise will interpret the findings differently. In approaching this literature review on the state-of-the-art design and manufacturing processes for custom manual wheelchair components, we acknowledge that the primary author’s background in mechanical engineering may shape the interpretation of existing research. To ensure a comprehensive and unbiased review, we employed a systematic approach for source selection and data analysis, using established criteria and methodologies to mitigate potential bias in our findings.

## Results

**Fig. 1:**
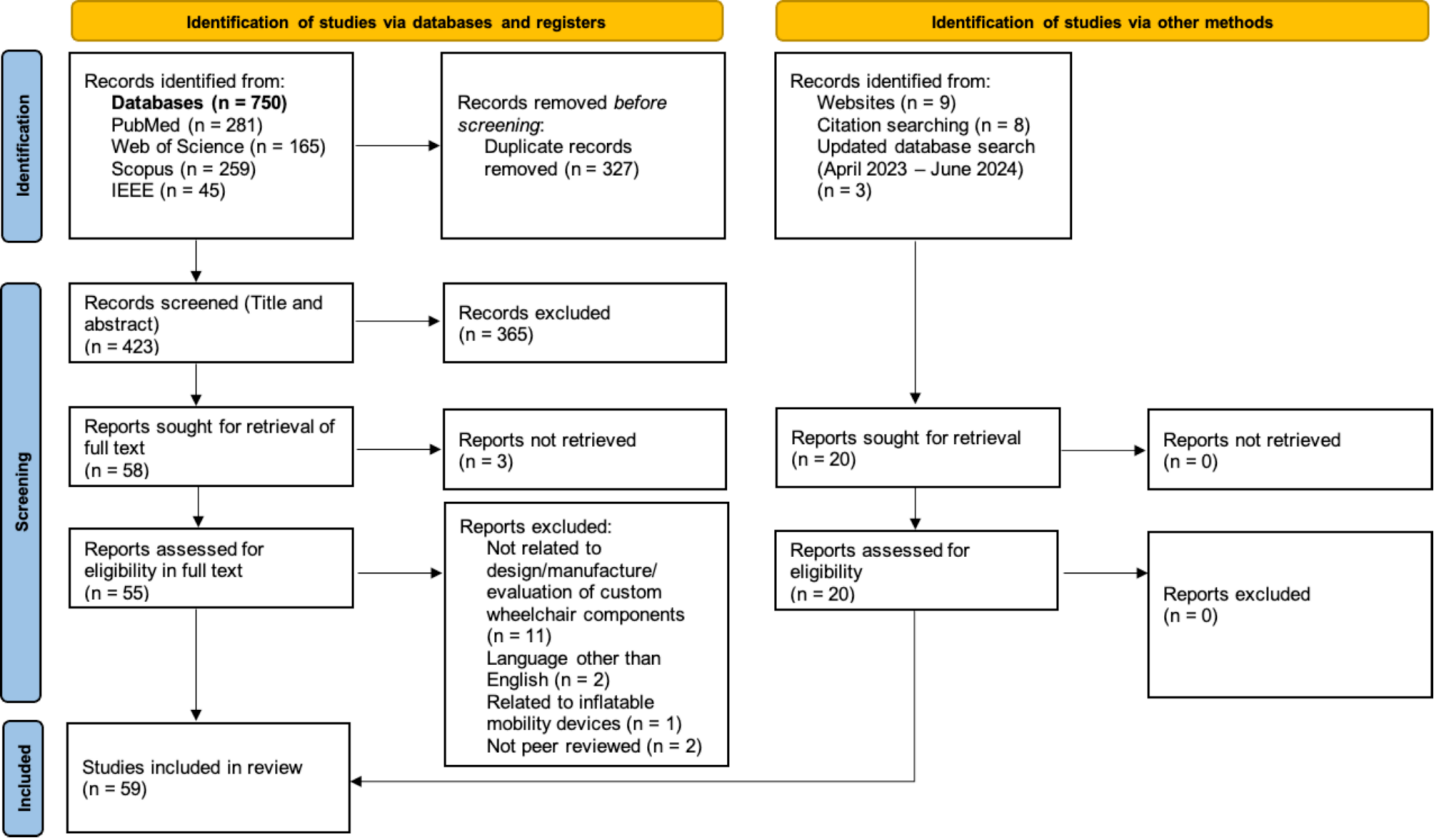
PRISMA flow diagram

The search strategy identified 750 articles, of which 327 were duplicates. After the titles and abstracts were examined on the basis of the inclusion and exclusion criteria, 365 articles were excluded. A total of 58 articles were selected for full-text review and screened for eligibility. However, the full texts of three articles could not be retrieved, as they were available only as conference abstracts. The full-text review excluded 16 articles for reasons such as not being related to the design/manufacturing/evaluation of customized wheelchair components, articles not being in English, articles related to inflatable mobility devices, and articles not being peer reviewed. More studies were identified through citation searching in included articles (*n* = 8), running an updated database search to capture new publications from April 2023-June 2024 (*n* = 3), and browsing through manufacturer websites (*n* = 9). Consequently, 59 articles were included in this review (Fig. 1).

### Main research areas

The research questions guided the classification of the included articles in the main research areas. The main research area was determined after reviewing the keywords and findings from the data extraction table. The first author performed this categorization, and the classification process and the identified research areas were then presented to the other authors to ensure consistency in categorization. This resulted in six categories (Table 2). Notably, some articles were classified into multiple categories because they focused on several aspects. Results from website browsing were used in relevant sections to support findings on state-of-the-art processes. The rest of the results section delves deeper into each category with the aim of understanding the current state-of-the-art processes in design and manufacturing.

**Table 2:** Categorization of articles based on the main research area

Research area	Number of articles	References
**Design of custom wheelchair components**		
Wheelchair frame	2	[19, 26]
Cushion	11	[27–37]
Back support	2	[19, 38]
**Manufacturing of custom wheelchair components**		
Wheelchair frame	2	[19, 26]
Cushion	13	[29–31, 33, 39–47]
Head support	1	[48]
**Design models for wheelchair customization**		
User needs assessment	3	[49–51]
Analytical model	1	[52]
Theoretical model	1	[53]
**Use of Computer based (CAx) tools**		
Design	13	[18, 19, 26, 36, 40, 42, 45, 53–58]
Manufacturing	4	[31, 42, 45, 55]
Analysis/Simulation	6	[34, 54, 55, 57, 59, 60]
**Evaluating effectiveness of custom components**		
Cushion	6	[39, 59, 61–64]
Wheelchair assembly	2	[65, 66]
**Customization impact and context**		
Benefits of customization	7	[6, 32, 65, 67–70]
Provision of custom wheelchairs	1	[71]
History of customization	1	[22]

### Customization impact and context

#### History of wheelchair customization

The concept of wheelchair customization emerged during the ultralight wheelchair revolution that occurred in the late 1970s. This was the time when wheelchair athletes began to modify their regular wheelchairs by relocating the axles to change the center of gravity. Such modifications unloaded the front castors, enabling the user to cover larger arcs with their arms along the hand rims, thus achieving greater speed. Other significant advancements during the revolution included the use of materials such as aluminum and titanium, the introduction of quick-release axles, adjustable rear wheel positions, and solid front castors. Consequently, the ultralight wheelchair revolution paved the way for the creation of custom-made wheelchairs that fit the individual user’s specific dimensions and preferences [22].

#### Benefits of wheelchair customization

Gardner [70] explored why older adults (aged 65+) customize their wheelchairs and identified three main motivations: fun, function, and fashion. Almost all the participants in this study referred to fun as the motivator for customizing their wheelchairs. This ‘fun’ was related to decorating their wheelchairs to express their interests, as well as making others engage with them through such additions. Interestingly, in the context of this study, the term “function” referred to how well the mobility device complemented the user’s specific needs, as opposed to the traditional focus on mechanical functionality. Finally, fashion customization was related to the user’s desire to make the wheelchair reflect their personal style. This included adding accessories, color themes, and decorations to the wheelchair [70]. Although these customizations were linked with positive health outcomes, studies have found that the user’s background (living at home or in an institutional setting) determines their possibility of having access to a custom wheelchair [67, 68]. Some experimental studies have also evaluated the effects of changing wheelchair parameters, such as wheelchair type [65], wheelchair mass, tyre pressure, and tyre type, on user mobility [69]. They have shown that customizing a wheelchair improves propulsion performance, ultimately leading to reduced physical strain.

#### Provision of custom wheelchairs

A time-motion study [71] looked at the provision of wheelchairs and seating systems with the aim of finding activities that can last for lengthy periods of time. They reported that the broad category of ultralightweight wheelchairs was associated with extended activity times, specifically assessment, order specification and assembly activities. The greater adjustability of these wheelchairs was identified as the main reason for these extended times.

### Design of customizable wheelchair components

#### Frame

A wheelchair frame, sometimes referred to as a chassis, can be folding or rigid and performs the essential function of holding all components together and supporting the user [32, 51]. Anthropometric measurements, functional requirements, and other user preferences are critical in designing a custom frame. Furthermore, frame angles can affect seat interface pressures as well as the distance between the user and the wheel, which affects the mechanics of propulsion [6]. The custom frame design can also reduce the weight of the wheelchair [18, 72]. Hence, it is critical that the frame be customized to the user.

**Fig. 2:**
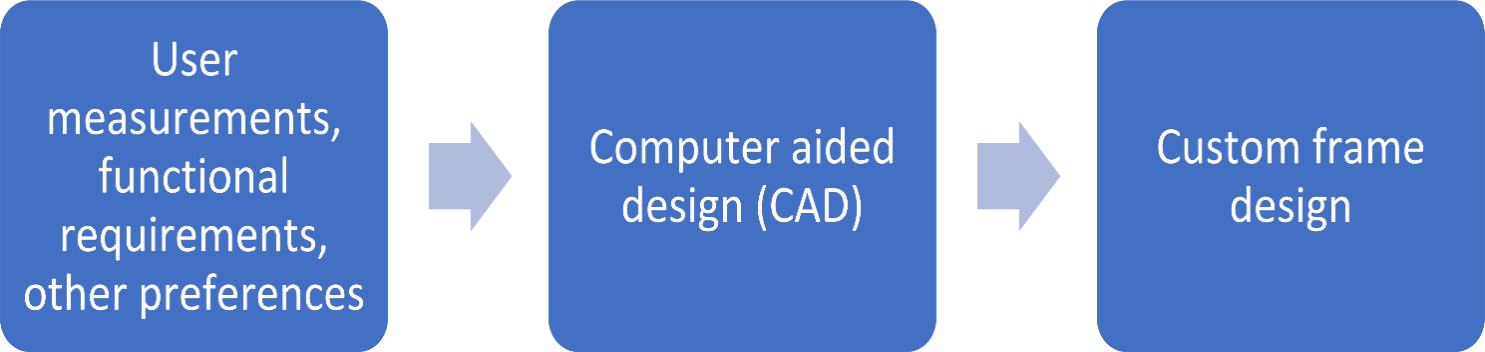
Design workflow for wheelchair frame customization

Two studies [19, 73] discussed the general design workflow followed for the design of a custom frame, which is shown in Fig. 2. Similar workflows were mentioned by some manufacturers (TiLite [74], RGK [75]). Both studies mentioned the use of a measuring/fitting chair to assist in recording user measurements. Using such fitting chairs, users can feel the seating position, verify driving behaviors, and discuss suitability with health professionals. To provide realistic feelings about the seating position, it was also suggested that the fitting chair be made with the same materials as the final chair.

#### Custom-contoured seating components

##### Cushions

Custom contoured seating is a seating system designed to fit the shape and size of an individual’s body. It is commonly used for people who spend extensive periods in a chair and are unable to shift their position. This type of seating is often placed on either manual or powered mobility bases [76]. The prolonged sitting times increases the risk of pressure injuries. The risk is even greater for wheelchair users with no sensation in the lower extremities [61]. These pressure injuries are a result of the higher seating interface pressures; therefore, custom-contoured cushions are essential for providing support for these users [39, 62]. In addition to wheelchair users, those with asymmetry in the pelvis, hips, or spine; low postural ability; and other movement disorders also use custom-designed seating [32]. Experimental studies in the 1990s indicated that customizing a cushion to the shape of the user resulted in lower seating interface pressures, thereby reducing the chances of pressure injuries [61]. This was the time when new techniques for the design and manufacturing of custom-contoured cushions were developed. Such cushions helped with pressure relief, posture control, and positioning [44].

The design of a custom-contoured cushion begins with capturing the contour of the user’s buttocks. Initially, these contours were obtained by measuring the deflection of a loaded flat foam cushion via a contour gauge [61] or by measuring the deformation of soft tissues [27]. These methods of capturing contours were later discovered to be costly, nonportable, and required complicated instrumentation, thus restricting their clinical application. As a result, interface pressure measurement using commercially available pressure mapping systems was proposed as a method to capture seat contours [28, 29, 33]. In addition to these methods, other forms of shape capture, such as vacuum consolidation and plaster casting, have also been used. However, these methods are costly, labor intensive, and not easily reproducible. As a result, recent methods have focused on digital shape capture via laser scanning and photogrammetry [31, 36, 40]. This is now becoming an industry standard and has already been adopted by manufacturers such as OttoBock [77], Invacare [78] and PRM Rehab [79]. Notably, all the studies in this section captured the seat contours under loaded conditions, where the buttocks and thighs were compressed due to gravity. As a result, a recent study looked at capturing seat contours in an unloaded (supine) position and reported that it can be a feasible method of capturing seat contours. These cushions were also lighter than other cushions. As this was a pilot study, the need for further research was identified to compare it with traditional shape capture methods and to use larger sample sizes [35].

Although contoured cushions can assist in reducing seating interface pressures, they cannot actively sense and respond to changing pressures. Therefore, a recent study by Robinson et al. [37] focused on creating a dynamic cushion that senses areas of high pressure and automatically actuates to relieve that pressure. They used a combination of resistive sensor arrays to sense changes in interface pressures and pneumatically actuated polymeric bladders that redistribute pressures from higher concentration areas. They also highlighted the possibilities of customization in the design of such cushions by changing bladder shapes and placement. The proposed design was tested to confirm its use in actively redistributing seating interface pressures.

##### Back support

Manual wheelchairs can have different types of back supports: planar, contour, or custom molded [12]. Like cushions, custom back supports distribute pressure evenly on the user’s back and provide postural support. Customizing back supports is crucial for individuals with spinal cord injuries (SCI) and other neurological conditions because standard rigid back supports may not accommodate certain fixed postural deformities. A study conducted by Crytzer et al. [38] utilized a laser scanner to capture the back shapes of users in two sitting positions, i.e., forward lean and upright, to determine if there were differences in the captured contour. No differences were found in the captured contour, negating the effect of the sitting position on the captured back contours. Another study by Barbareschi et al. [19] developed a device called the dimensional information measurement system (DIMS) to assist the clinician in measuring 3D points in space and to capture the contour of the user’s back. These data were then fed to a parametric model that created the custom back shape. Finally, all the techniques for cushion design discussed in the literature review by Nace et al. [42] can also be applied to back supports.

### Manufacturing of customizable wheelchair components

#### Frame

Two studies discussed the manufacturing of custom frames. The first [73] mentioned the use of adhesive joining techniques to join carbon fiber tubes. They also discussed the importance of having closer tolerances for manufactured frames. This was achieved via an adjustable jig that links with the 3D model of the wheelchair, ensuring the exact transfer of user measurements to the manufactured frame. The second study [19] discussed the use of 3D printing for creating custom frames in low- and middle-income countries. The tube lengths and joint dimensions were automatically updated via a parametric CAD model, and the resulting files were then used for 3D printing.

#### Custom-contoured seating components

##### Cushions

Initially, contoured cushion manufacturing relied on a manual approach that involved hand carving the seat contour. This method relied heavily on the technician’s skills, and making any adjustments to the contour requires multiple fittings [45]. A state-of-the-art literature review on custom-contoured seat manufacturing conducted by Nace et al. [42] mentioned several other manufacturing methods, such as foam-in-place seating (FIPS) [80], adjustable micromodular seating (AMMS) sheets [81], vacuum forming [43] and computer numerical control (CNC) foam milling [31]. Among these techniques, CNC foam milling has emerged as the current state-of-the-art process because of its minimal reliance on manual labor. This process relies on the use of software tools to process the captured contour of the user and generate tool paths to guide the cutting tool. The accuracy of manufacturing then depends on parameters such as the tool type, milling speed, milling direction, and feed rate, all of which depend on the type of foam being milled [31]. The state-of-the-art review also recognized the potential of additive manufacturing approaches to offer enhanced customization options, emphasizing the need for further research on their application in custom-contoured seating. In addition, they discussed the importance of manufacturing in-house in a clinical setting to reduce user wait times and highlighted the need for further research to standardize a scan-to-print method. As a result, a recent study by Polydorides and Rogers [47] demonstrated approaches using 3D printing for customizing cushions. This customization was achieved by varying the infill percentage of the cushion on the basis of the pressure distribution at the seating interface. Interestingly, all the approaches discussed in their study needed minimum to no CAD skills, making them suitable for use in a clinical environment. The need for further research on the user testing of these cushions was highlighted.

##### Head support

Methods of customizing cushions and backrests are standardized, but such processes do not exist for head supports. As a result, Howard et al. [48] investigated the use of additive manufacturing for the production of custom head supports. The proposed workflow included 3D scanning, CAD, and additive manufacturing and could be used by seating specialists to fabricate custom head supports. They also validated this process by comparing failure mechanisms with those of commercially available head supports. Furthermore, the need for research was proposed to refine design and manufacturing parameters, understand the financial implications of adopting such systems, and incorporate design automation, which would make the process feasible for use in a clinical environment.

### Design models for wheelchair customization

#### User needs assessment

Traditional design methods fail to satisfy user requirements and may also lead to extended design times when they are applied to wheelchair customization. The development of new models for customization can help mitigate these issues [50]. Wheelchair prescriptions involve multidisciplinary teams consisting of health professionals, rehabilitation engineers, and technicians. These teams have the complex task of identifying user needs and require reliable tools to guide the decision-making process [49]. This is where existing frameworks for prioritizing user needs have been applied in the context of wheelchair customization.

First, Logan and Radcliffe [49] explored the use of quality function deployment (QFD) to capture user needs and develop engineering requirements for manufacturing customized wheelchair seating in a seating clinic context. They used the House of Quality (H_of_Q) matrix to visualize the interrelationships between user and engineering requirements. Considering the wider group consisting of users, family, carers, professional service providers, and cross-functional teams was identified as essential for the success of customization. Although this technique is useful, time and personnel constraints in the seating clinic limit the depth of analysis. Hence, the advice was to use it occasionally for users with complicated needs. Second, Yuan and Guan [50] used the analytic hierarchy process (AHP) and Kano model to classify wheelchair functions and user needs and determine their priorities. They claimed that using such methods can lead to improved user satisfaction in the customization process.

#### Analytical model

A kinematic model for customizing wheelchair design was developed by Shyu et al. [52]. This model was based on optimizing the geometrical configuration of the wheelchair by using mechanical analysis concepts from the field of solid mechanics to reduce upper limb injuries. To calculate this optimum configuration, they simulated the human chair system as a planar five-bar linkage model between the upper limb and hand rim. The study revealed that an obvious advantage of using such a model was the rapid customization of the wheelchair configuration, which would lead to better user device fit and a reduction in design and manufacturing time. They proposed that validating such models could lead to the development of software systems to calculate wheelchair size and seat position on the basis of the user’s upper limb measurements.

#### Theoretical model

Joergensen et al. [53] proposed a theoretical model based on the concept of mass customization to assist product designers in determining the level of customization. This model proposes four levels of customization: structure, performance, experience, and learning.

- Structure level: This is the lowest level of customization, which focuses on offering components and determining which components are customized and manufactured in-house and which are standard and outsourced or bulk purchased.
- Performance level: This level focuses on considerations regarding the customized product’s performance. The requirements identified at this level can further constrain the structural level.
- Experience level: This level relates to intangible product attributes that are related to the user’s dreams and emotions, that is, personalization.
- Learning level: This level is related to the transfer of product knowledge to the user. It can include websites or tutorials that can assist the user in better understanding the product.

Each of these levels assists in identifying design requirements, which can be interdependent on other levels. Hence, ideally, a good balance of all levels must be achieved. They used a custom manual wheelchair as a case study and demonstrated the use of this model.

### Use of computer-based (CAx) tools in customizing wheelchair components

CAx tools can improve quality and productivity and reduce delivery times [55]. They have been used to assist in different stages of the product development lifecycle, such as design, manufacturing, and analysis.

#### Design

The earliest use of computer-based tools in the rehabilitation industry was mentioned by Rogers and Fulton [54], who also developed a prototype system to model the human body for assisting with wheelchair fitting. This helped in calculating stability relationships for the chair and the user as a combined entity. Other studies have also used these tools for the modification of 3D solid models on the basis of user measurements [18, 19, 58, 73], performing interference checks on customized designs [73], converting and modifying 3D scans to editable solid models [31, 35, 36, 57, 59], comparing 3D scans [40], and creating solid models via MRI images [34]. All the uses of 3D scanning were related to custom-contoured cushions and back supports, indicating that this is a state-of-the-art process for custom-contoured seating components. Finally, a recent study focused on creating digital workflows that ease the process of designing contoured cushions without increasing costs, with the aim of making computer-based design tools more accessible to diverse user bases [36].

Another important thing to note is the increasing use of product configurators in the design of custom wheelchairs. These tools primarily support users in the wheelchair configuration process [53]. Several manufacturers, such as Sunrise Medical (Quickie wheelchairs) [82], Kuschall [83], and Permobil (Progeo wheelchairs) [84], have developed configuration tools, with some able to export the configured model in augmented reality.

#### Manufacturing

The use of computer-based tools in manufacturing can be applied in industrial or clinical contexts [55]. Research by Lemaire et al. [45] has shown that the use of these tools can greatly reduce manufacturing times and post manufacturing modifications. The adoption of such systems depends on local economic factors, including device and material costs. Furthermore, other studies have employed computer-based tools to determine tool paths for CNC milling of foam cushions [31, 42].

#### Analysis

Computer-based simulation and analysis tools provide a virtual environment in which designers can evaluate the performance and functionality of different components before physically prototyping them. Rogers and Fulton [54] documented the earliest use of these tools in rehabilitation engineering. They used simulation tools for the structural analysis of wheelchairs, indicating that they could optimize component weights while maintaining structural integrity. Rogers et al. [60] employed finite element analysis (FEA) to perform virtual crash tests to ensure compliance with wheelchair standards. They also highlighted that while these virtual models can provide valuable insights, it is crucial to validate them through physical testing before they are used to make decisions. They also concluded that such models cannot completely replace physical testing in the development and validation process of custom manual wheelchair components.

FEA has also been used in custom-contoured seating to evaluate the mechanical interaction between the cushion and wheelchair user [57] and investigate the internal stress distribution between soft tissues to evaluate the performance of custom-contoured cushions in static sitting [59] and propulsion conditions [34]. These studies developed user-specific simulation models and experimentally validated them, which supports their use in the analysis of custom-contoured cushions. However, the need for further research to develop more realistic models for analysis was highlighted.

### Evaluating the effectiveness of custom wheelchair components

The customization of wheelchair components can significantly improve users’ comfort and social participation. This section summarizes the metrics that have been used to assess the effectiveness of custom components. These metrics can be used to evaluate individual components or the wheelchair assembly as a whole. First, with respect to individual components, the functionality of custom-contoured seating components has been evaluated in all studies via interface pressure measurements. Furthermore, some studies have also evaluated comfort subjectively via questionnaires [39, 59]. For wheelchair assembly, Beekman et al. [66] evaluated functionality using speed, distance traveled, and the oxygen cost per distance traveled. Finally, a study by Andrews et al. [65] used the wheelchair propulsion test (WPT) to compare a customized wheelchair to other standard options.

These metrics provide valuable insights into the effectiveness of custom wheelchair components in terms of functionality and comfort. Overall, the evaluation of custom wheelchair components is crucial in determining their effectiveness in enhancing mobility and improving users’ quality of life.

## Discussion

The primary aim of this review was to understand the current state of research on the design and manufacturing of customizable wheelchair components. To achieve this goal, we used a state-of-the-art review methodology to conduct the review systematically. The articles in this review were classified into six categories: (1) design of custom wheelchair components, (2) manufacturing of custom wheelchair components, (3) design models for wheelchair customization, (4) use of computer-based (CAx) tools, (5) evaluation of the effectiveness of custom components, and (6) customization impact and context.

Starting with the design of custom components, custom-contoured cushions have been studied the most in the literature (*n* = 11), and a small number of studies have focused on wheelchair frames (*n* = 2) and backrests (*n* = 2). A possible explanation for the focus on custom-contoured cushions could be the advances in the field of custom orthoses, which have a design process similar to that of contoured cushions [36]. Research on other similar components, such as bicycle frames, could help progress research on wheelchair frames. An interesting finding with respect to frame design was the use of fitting/measuring chairs, also referred to as wheelchair simulators [19]. The use of such measuring chairs provides several advantages, such as the ability to identify the exact seating position of the custom chair before manufacturing and the reduction in errors while recording the user’s measurements [19, 73]. However, to establish them as a formal assessment tool, more studies are needed to confirm their usability. Furthermore, there are potential applications of 3D scanning in measuring wheelchair fit as well as capturing dimensions, with examples available from some organizations [85–87]. As such, more empirical studies are needed to establish state-of-the-art in measuring wheelchair fit.

In terms of manufacturing custom components, a similar focus on custom-contoured cushions was observed (*n* = 13), with a small number of studies looking at frames (*n* = 2) and headrests (*n* = 1). Compared with the field of custom orthoses, where the focus has been on making the manufacturing process easier and quicker for orthopedists [88], recent research on manufacturing contoured cushions has also focused on making advanced manufacturing feasible in a clinical environment by standardizing 3D scanning workflows for milling and additive manufacturing [36]. Such technologies can potentially reduce the lead time for custom-contoured cushions. Finally, additive manufacturing is being explored as a potential technique for producing complex frames and headrests.

Research on design models for customizable components has focused on applying existing frameworks for user needs assessment (*n* = 3), whereas single occurrences have been observed for analytical (*n* = 1) and theoretical models (*n* = 1). All studies using existing frameworks for needs assessment have demonstrated their applicability in evaluating wheelchair concepts. Designers and rehabilitation engineers could use these as resources while evaluating their design concepts for new wheelchairs. In addition, analytical models can be developed into software tools that enable rapid customization of components, ultimately reducing design costs. Finally, the theoretical model, which is based on the concept of mass customization, can be used to assist in decision-making regarding the level of customization for manual wheelchairs. This ensures that the customization of components does not drastically increase costs.

Many articles (*n* = 23) reported the use of computer-based tools to customize wheelchair components. Within this category, the design subcategory had the highest number of contributions (*n* = 12), pointing toward the rising trend of digital tools in the design of custom wheelchair components. The rise in the use of these tools can be attributed to the availability of portable computing power and the advent of rapid prototyping and flexible manufacturing techniques [18]. The increasing use of product configurators to assist stakeholders in visualizing custom designs must also be noted. Such tools can ensure active user participation in the design process, leading to better outcomes. Further studies could look at the usability of these configurators in decision-making regarding customizations. One study [36] mentioned that the requirement to have a CAD specialist on the team could be seen as a barrier to adopting advanced digital tools in the clinical environment. Hence, complete or partial automation of digital workflows to make them suitable for use in the clinical environment is a topic of interest for further research. Some studies (*n* = 6) also used FEA tools to understand stress distributions in components and validated such models through experiments. These studies could be used as resources to develop realistic and applicable models by generating guidelines for applying constraints and loading conditions for different components.

The literature does not recommend industry-standard software, so further studies should compare software tools on the basis of their ease of use, compatibility with 3D scanning equipment, core manufacturing processes, and costs, to name a few. Further advances in additive manufacturing, artificial intelligence, machine learning, and computer based tools are expected to enable the production of even more complex and customized components [18]. Therefore, future research should continue to investigate the use of emerging technologies in the customization process and evaluate their impact on user centered outcomes, performance and aesthetics of wheelchair components. Several outcome measures as discussed by Robertson et al. [89], could assist in such evaluations. Furthermore, activities in the provision process of wheelchairs are influenced by geographic location as well as business practices followed by manufacturers [71]. Hence, more research on design and manufacturing in different contexts (developed countries, developing countries, large manufacturers, and small manufacturers) can lead to context-specific innovations in the design and manufacturing of customizable wheelchair components.

The lack of evidence on the impact of providing a properly fitted custom wheelchair must also be noted. This general lack of evidence has already been highlighted by prior studies on wheelchair provision [4, 10]. Although this review identified only two studies evaluating the impact of custom wheelchairs on propulsion performance [65, 66], there are many more potential outcome measures to consider. More research is needed to generate robust evidence on the life-changing consequences that can result from obtaining a properly fitted custom wheelchair. The outcome measures discussed by Robertson et al. [89] could prove helpful in this regard.

### Use of terminologies

We found that in the realm of manual wheelchairs, words *personalization* and *customization* have often been used interchangeably in the literature, with a small number of articles (*n* = 5) using word *personalization*. However, within the scope of this review, we aim to establish a clearer distinction. *Customization* involves the modification of wheelchair components on the basis of user measurements or contours. In contrast, *personalization* is associated with the addition of accessories or the modification of the appearance of components according to user preferences. Another essential thing to know is the distinction between *custom*/*made-to-measure* and *bespoke* wheelchairs. Although no definitions are currently available for these terms in the context of wheelchairs, analogies from the garment industry could be used to explain them. Here, *bespoke* refers to garments designed from scratch, whereas *made-to-measure* refers to garments made via a standardized pattern [90]. Similarly, *custom* and *made-to-measure*, as used by some manufacturers [91, 92], relates to the wheelchair frame being made according to user measurements. For these wheelchairs, the base design is tailored according to the user dimensions. Finally, *bespoke* wheelchairs are made according to user dimensions but not on the basis of any predefined design. However, in the literature, these words are still used interchangeably. Hence, attention must be given to terminologies while studying the literature on custom wheelchairs.

In the context of customisation of manual wheelchairs, it is important to agree a definition that is related to both the wheelchair and the ISO standards; and potentially agree a hierarchy. For example, Individualization can relate to updating a base design to fit the user, but customisation means customized to an individual’s own unique postures/contours. Bespoke on the other hand, could either be interchangeable with customization, or refer only to aesthetics. This remains an area of contention and requires further work as to harmonization of language. This further is important when defining further empirical evaluation.

## Limitations

We acknowledge that to understand the state of the art in this field, one needs to go beyond published literature, and look at manufacturers websites or conduct workshops with them, as manufacturers do not publish their techniques in academic literature. While some manufacturers are mentioned in this review, benchmarking their design and manufacturing practices was not the primary focus. This also leads to the fact that there is much more customization happening than what is reported in this review. Furthermore, no distinction was made between studies in high- and low-income countries or different wheelchair provision models, which can affect the design and manufacturing processes. Hence, these results are not generalizable across contexts. In addition, the overlap in the definitions of ultralight and custom wheelchairs could also be seen as a limitation. This review did not consider the customization of components in other wheelchair categories, where there might be different approaches used for customization, such as in folding frame wheelchairs. Furthermore, it is important to note that although custom seating (inclusive of cushions, backrests, headrests) is discussed in this review, due to the focus on all customizable components of a manual wheelchair, contoured seating is mostly prescribed for power wheelchair users and manual tilt in space wheelchair users. Ultralight wheelchair users would normally be prescribed with off the shelf customizable seating components.

## Conclusion

The importance of assistive technology customization cannot be overstated, especially in the case of manual wheelchairs. Customization allows users to make product adaptations and aesthetic changes, which in turn reduces device abandonment and increases user participation and ownership. This literature review systematically categorized the existing body of research on the design and manufacturing of custom manual wheelchair components, identified current practices, and suggested areas for further research.

The review revealed that while there is a significant amount of literature on the design and manufacturing of custom-contoured cushions, studies focusing on other components or wheelchair customization as a whole are lacking. Technological advances in the past decade have seen the move from manual, time-consuming customization processes to digital workflows with the support of design tools. Advanced technologies such as 3D scanning, parametric modeling, product configuration systems, and finite element analysis have emerged as tools for enhancing the performance and aesthetics of wheelchair components. Additive manufacturing has been used to produce wheelchair frames and headrests, and its application to other components is on the horizon. By continuing to explore and develop these innovative approaches, researchers, manufacturers and health professionals can work towards ensuring that all users have access to appropriately customized, high-quality manual wheelchairs that promote their independence, social inclusion, and overall quality of life.

## Data Availability

No datasets were generated or analysed during the current study.

## Abbreviations

kg: Kilogram, a weight measurement unit
WHO: World health organization
CAD: Computer-aided design
PRISMA: Preferred reporting items for systematic reviews and meta-analyses
CAx tools: Computer-based tools
SCI: Spinal cord injury
DIMS: Dimensional information measurement system
FIPS: Foam-in-place seating
AMMS: Adjustable micromodular seating
CNC: Computer numerical control
QFD: Quality function deployment
H_of_Q: House of quality
AHP: Analytic hierarchy process
FEA: Finite element analysis
WPT: Wheelchair propulsion test
